# Nomograms incorporated serum direct bilirubin level for predicting prognosis in stages II and III colorectal cancer after radical resection

**DOI:** 10.18632/oncotarget.11424

**Published:** 2016-08-19

**Authors:** Qunfeng Zhang, Xiaowei Ma, Qunhuan Xu, Juanxiu Qin, Yanhua Wang, Qian Liu, Hua Wang, Min Li

**Affiliations:** ^1^ Department of Laboratory Medicine, The Fifth People’s Hospital of Shanghai, Fudan University, Shanghai, China; ^2^ Department of Laboratory Medicine, Renji Hospital, School of Medicine, Shanghai Jiaotong University, Shanghai, China

**Keywords:** colorectal cancer, direct bilirubin, surgery, survival analysis

## Abstract

An elevated serum bilirubin has been reported to be associated with a reduced risk of some cancer; however, the prognostic significance of serum bilirubin in colorectal cancer wasn’t fully understood. The purpose of this study was to evaluate whether serum bilirubin could predict the prognosis of patients in stages II and III colorectal cancer. A retrospective cohort of 986 patients with colorectal cancer who received surgical resection between January 2005 and December 2010 was included in the study. Levels for serum bilirubin were obtained from medical records. Survival analysis was used to evaluate the predictive value of bilirubin. Serum direct bilirubin (DBIL) was validated as a significant prognostic factor by univariate cox regression test for both overall survival (OS) and disease free survival (DFS) (*P* < 0.05). X-tile program identified 3.6 as optimal cutoff values for DBIL in terms of OS and DFS. Patients were then divided into DBIL high (DBIL ≥ 3.60 μmol/l) and low group (DBIL < 3.60 μmol/l) according to the optimal cutoff. High DBIL had higher percentage of lymph node metastasis and lymphovascular invasion as compared with low DBIL levels (*P* < 0.05). Multivariate cox regression analyses confirmed that high DBIL level was an independently prognostic factor for both OS (HR: 1.337, 95% CI: 1.022–1.748, *P* = 0.034) and DFS (HR: 1.312, 95% CI: 1.049–1.643, *P* = 0.018). In addition, nomograms on OS and DFS were established according to all significant factors, and c-indexes were 0.715 (95% CI: 0.683–0.748) and 0.704 (95% CI: 0.678–0.730), respectively. Nomograms based on OS and DFS can be recommended as practical models to evaluate prognosis for CRC patients.

## INTRODUCTION

Colorectal cancer (CRC) is the third most commonly diagnosed cancer and the third leading cause of cancer death world. In 2016, approximately 70,820 new cases and 26,020 CRC-related deaths were estimated in United States [1]. In China, the incidence of CRC has been increasing in recent years as living conditions improve and eating habits change. Surgical resection remains the only curative treatment opinion for CRC [2].

There are many known risk factors for predicting survival after surgical resection, including marital status, age, lymph node status, systemic inflammation, et al. [3–6]. Among them, the most significant risk factor is tumor-node-metastasis (TNM) staging. However, CRC is a heterogeneous disease, the current staging system is not precise for predicting patient outcomes because the prognosis varies even in patients with the same disease stage. Additional parameters need to be defined to better identify prognostic factors for patients, to allow tailored therapies [7].

Serum bilirubin, regarding as end product of heme metabolism, has been considered to have no physiological function. However, in recent years, some experimental and clinical researches have demonstrated that serum bilirubin has several protective effects, including potent antioxidant, anti-inflammatory and anticancer activities [8–10]. The inverse association between bilirubin and cancer risk has been observed in breast cancer [11], lung cancer [12] and colorectal cancer [10, 13]. Elevated serum bilirubin levels also are associated with improved survival in patients with curatively resected non-small-cell lung cancer [14]. Patients of breast cancer with higher total bilirubin level had a nearly 40% reduction in the risk of death [15]. Conversely, severe jaundice decreases long-term survival after pancreaticoduodenectomy for pancreatic adenocarcinoma [16]. Increased serum direct bilirubin level was associated with lymph node metastasis and poor prognosis in rectal cancer patients [8], however, in their study, only patients with rectal cancer were included, the primary endpoint only included the overall survival (OS), and patients with stage IV disease also included.

This study aimed to assess the ability of serum bilirubin level to predict survival outcome in patients with CRC after radical resection. There are many studies in which the development of nomograms leads to a successful application for oncology prognostics. Nomograms for predicting follow-up outcome for CRC are scarce [6]. We further develop nomograms to investigate the prognostic role of serum bilirubin level in patients with CRC.

## RESULTS

### Patients’ baseline characteristics

A total of 986 patients with stage II and stage III CRC were recruited for this study, including 379 patients with stage II and 607 patients with stage III disease. 357 (36.2%) patients suffered recurrences and 225 (22.8%) died by the date of the most recently scheduled follow-up. Table 1 summarizes the baseline characteristics of included patients. The mean age was 57.69 years with an SD of 12.18. There were more male patients than female patients, with a male/female ratio of 1.34.The median value of total bilirubin (TBIL), direct bilirubin (DBIL), indirect bilirubin (IDBIL) were 11.04 (range 2.70–66.50) μmol/l, 3.54 (range: 1.00–12.30) μmol/l, 7.50 (range: 1.70–54.20) μmol/l, respectively.

**Table 1:** Baseline of patients with colorectal cancer treated with surgical resection

Variable	DBIL group					
	Low		High			
	*N*	%	*N*	%	χ^2^	*P* value
Sex					0.683	0.408
Male	326	56.0	237	58.7		
Female	256	44.0	167	41.3		
Age					0.492	0.483
0	324	55.7	234	57.9		
> 60	258	44.3	170	42.1		
Primary site					0.649	0.421
Colon	286	49.1	188	46.5		
Rectum	296	50.9	216	53.5		
Grade					0.201	0.905
High/ Moderate	404	69.4	281	69.6		
Poor/ Anaplastic	141	24.2	100	24.8		
Unknown	37	6.4	23	5.7		
T stage					2.870	0.238
T1–2	81	13.9	68	16.8		
T3	301	51.7	215	53.2		
T4	200	34.4	121	30.0		
N stage					14.934	0.001
N0	245	42.1	134	33.2		
N1	199	33.2	126	31.2		
N2	144	24.7	144	35.6		
No. of LNs					3.736	0.053
< 12	157	27.0	132	32.7		
≥ 12	425	73.0	272	67.3		
Lymphovascular invasion					4.969	0.026
Negative	415	71.3	261	64.6		
Positive	617	28.7	143	35.4		
Perineural invasion					0.230	0.632
Negative	464	79.7	317	78.5		
Positive	118	20.3	87	21.5		

### Prognostic value of DBIL

**Figure 1:**
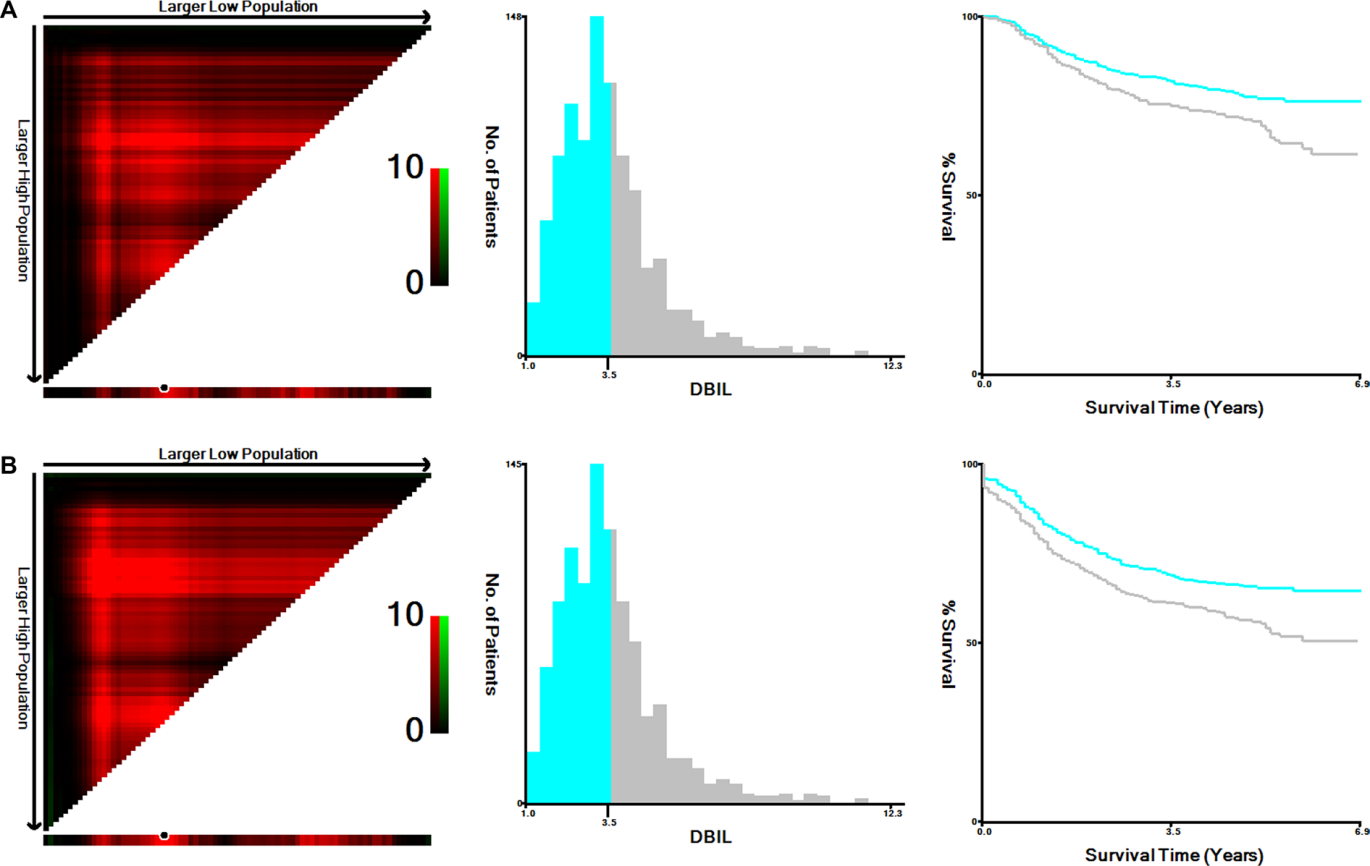
X-tile analyses of 5-year OS and DFS were performed using patients’ data to determine the optimal cut-off value for DBIL The sample of CRC patients was equally divided into training and validation sets. X-tile plots of training sets are shown in the *left panels*, with plots of matched validation sets shown in the *smaller inset*. The optimal cut-off values highlighted by the *black circles* in *left panels* are shown in histograms of the entire cohort (*middle panels*), and Kaplan-Meier plots are displayed in *right panels*. *P* values were determined by using the cut-off values defined in training sets and applying them to validation sets. The optimal cut-off value for DBIL in terms of OS and DFS happens to be 3.6 μmol/l.

We first treated TBIL, DBIL,IDBIL as a continuous variable, and only DBIL was validated as a significant prognostic factor by univariate cox rgression test for both OS and disease free survival (DFS) (*P* < 0.05) (Table 2). We then used X-tile program to determine the optimal cut-off values for DBIL in terms of OS and DFS, which happens to be 3.6 μmol/l for both OS and DFS (Figure 1). Patients were then divided into DBIL high (DBIL ≥ 3.60 μmol/l) and low group (DBIL < 3.60 μmol/l) according to the optimal cutoff. Patients in high DBIL group had higher percentage of lymph node metastasis (N1 and N2 stage) and lymphovascular invasion as compared with those patients in DBIL low group (Table 1).

**Table 2:** Univariate cox rgression test of TBIL, DBIL,IDBIL on survival outcome in stage II and stage III CRC patients after radical resection

Variable	HR	95% CI	*P* value
**OS**			
TBIL	0.917	0.789–1.067	0.263
DBIL	1.112	1.036–1.194	0.003
IDBIL	0.906	0.738–1.111	0.342
**DFS**			
TBIL	1.018	1.000–1.037	0.055
DBIL	1.097	1.033–1.164	0.003
IDBIL	1.017	0.992–1.044	0.182

Aside from DBIL, patients in elder group (*P* = 0.023), with poor differentiated caicinoma (*P* < 0.001), with advanced T stage (*P* = 0.001) and N stage (*P* < 0.001), presented with lymphovascular invasion (*P* < 0.001) and perineural invasion (*P* = 0.001) shared a worse 5-year OS (Table 3). In addition, patients with poor differentiated caicinoma (*P* < 0.001), with advanced T stage (*P* < 0.001) and N stage (*P* < 0.001), with less than 12 lymph node retrieval (*P* = 0.005), presented with lymphovascular invasion (*P* < 0.001) and perineural invasion (*P* < 0.001) suffered a shorter 5-year DFS (Table 4).

**Table 3:** Univariate and multivariate survival analyses for evaluating the influence of serum direct bilirubin on OS in stage II and III colorectal cancer

Variable	5-year OS	Univariate analysis		Multivariate analysis	
		Log rank χ^2^ test	*P*	HR (95% CI)	*P*
Sex		0.388	0.533		
Male	73.4%				
Female	74.9%				
Age		5.170	0.023		0.004
< 60	76.9%			Reference	
≥ 60	71.0%			1.493 (1.140–1.955)	
Primarysite		0.013	0.911		
Colon	74.3%				
Rectum	73.8%				
Grade		21.388	< 0.001		0.267
High/ Moderate	77.9%			Reference	
Poor/ Anaplastic	64.1%			1.283 (0.942–1.747)	0.114
Unknown	69.6%			1.214 (0.733-2.010)	0.452
T Stage		13.375	0.001		0.042
T1–2	86.1%			Reference	
T3	73.2%			1.744 (1.054–2.884)	0.030
T4	69.8%			1.950 (1.159–3.280)	0.012
N stage		92.978	< 0.001		< 0.001
N0	88.0%			Reference	
N1	75.3%			1.944 (1.312–2.897)	0.001
N2	54.1%			3.469 (2.318–5.190)	< 0.001
No. of LNs		1.836	0.175		
< 12	70.9%				
≥ 12	75.7%				
Lymphovascular invasion		64.976	< 0.001		0.003
Negative	82.0%			Reference	
Positive	57.7%			1.568 (1.163–2.112)	
Perineural invasion		11.873	0.001		0.899
Negative	77.0%			Reference	
Positive	64.1%			1.020 (0.747–1.393)	
DBIL		9.213	0.002		0.034
Low	77.0%			Reference	
High	70.0%			1.337 (1.022–1.748)	

NI: not included in the multivariate survival analysis.

**Table 4:** Univariate and multivariate survival analyses for evaluating the influence of serum direct bilirubin on DFS in stage II and III colorectal cancer

Variable	5-year DFS	Univariate analysis		Multivariate analysis	
		Log rank χ^2^ test	*P*	HR (95% CI)	*P*
Sex		0.335	0.563		
Male	61.3%				
Female	64.4%				
Age		0.082	0.774		
< 60	63.6%				
≥ 60	61.6%				
Primarysite		0.130	0.719		
Colon	63.7%				
Rectum	61.0%				
Grade		19.782	< 0.001		0.345
High/ Moderate	66.3%			Reference	
Poor/ Anaplastic	53.4%			1.200 (0.923–1.562)	0.173
Unknown	56.7%			1.208 (0.775–1.883)	0.405
T Stage		15.411	< 0.001		0.002
T1–2	77.3%			Reference	
T3	61.3%			1.631 (1.099–2.422)	0.015
T4	57.7%			2.049 (1.360–3.088)	0.001
N stage		112.864	< 0.001		< 0.001
N0	80.5%			Reference	
N1	59.9%			2.002 (1.471–2.727)	< 0.001
N2	39.9%			3.212 (2.322–4.443)	< 0.001
No. of LNs		7.792	0.005		0.004
< 12	55.5%			Reference	
≥ 12	65.6%			0.710 (0.563–0.897)	
Lymphovascular invasion		46.655	< 0.001		0.279
Negative	70.3%			Reference	
Positive	46.4%			1.150 (0.893–1.480)	
Perineural invasion		37.396	< 0.001		0.010
Negative	67.0%			Reference	
Positive	45.2%			1.403 (1.086–1.811)	
DBIL		10.250	0.001		0.018
Low	66.5%			Reference	
High	57.1%			1.312 (1.049–1.643)	

NI: not included in the multivariate survival analysis.

The variables significant in univariate log-rank test were incorported into multivariate cox regression analysis.The results confirmed that high DBIL level was an independently prognostic factor for both OS (HR:1.337, 95% CI:1.022–1.748, *P* = 0.034) and DFS (HR:1.312,95% CI: 1.049–1.643, *P* = 0.018) in patients with stage II and stage III CRC after surgical resection (Tables 3 and 4).

### Development and validation of nomograms for predicting prognosis of CRC patients

**Figure 2:**
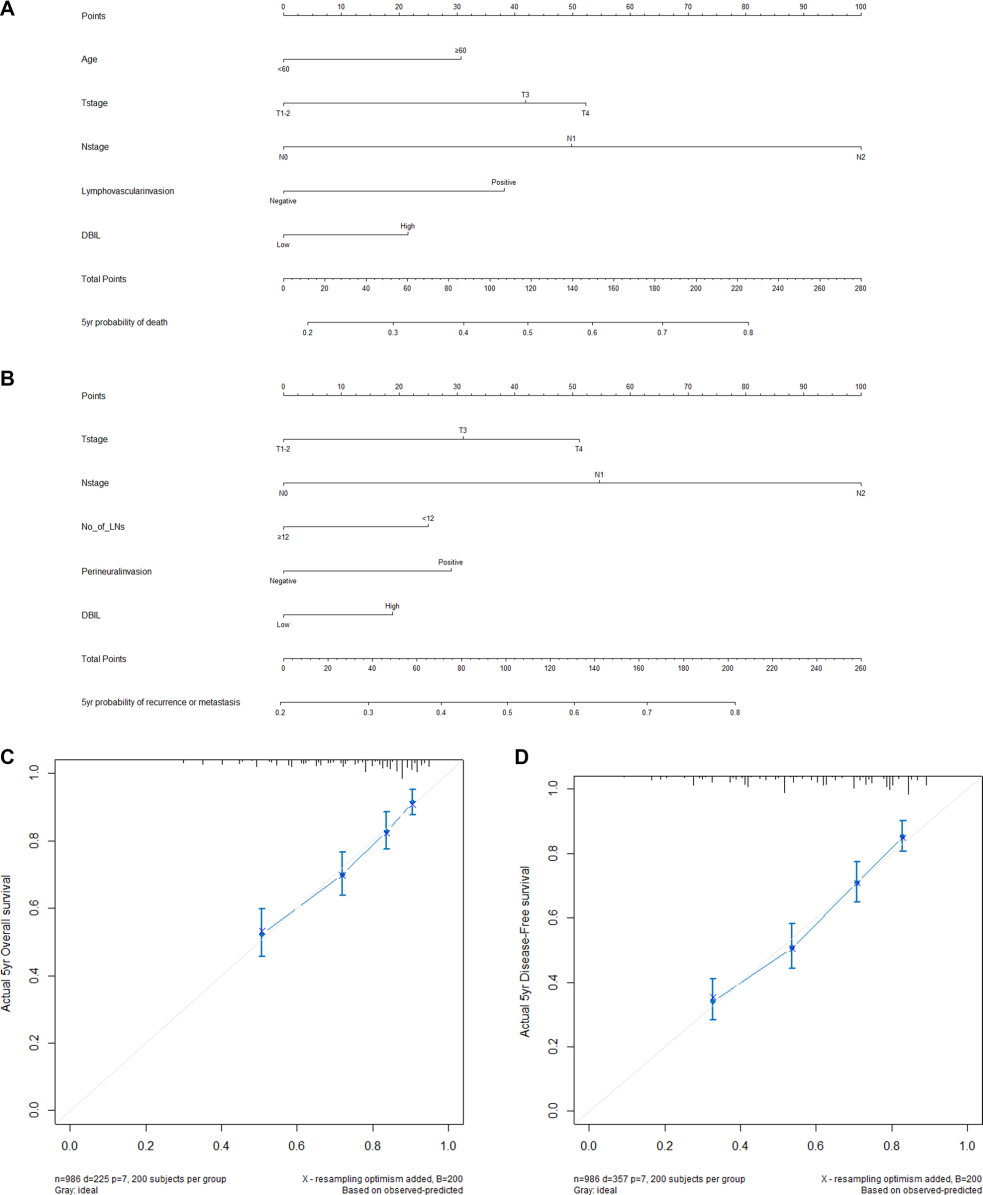
Nomograms conveyed the results of prognostic models using clinicopathological characteristics and pretreatment inflammatory biomarkers to predict OS (A) and DFS (B) of patients with CRC Nomograms can be interpreted by summing up the points assigned to each variable, which is indicated at the top of scale. The total points can be converted to predicted 5-year probability of death and recurrence or metastasis for a patient in the lowest scale. The Harrell’s c-indexes for OS and DFS prediction were 0.715 (95% CI: 0.683–0.748) and 0.704 (95% CI: 0.678–0.730), respectively. Calibration curves for 5-year OS (**C**) and 5-year DFS (**D**) using nomograms with clinicopathological characteristics and pretreatment inflammatory biomarkers are shown. The *x*-axis is nomogram-predicted probability of survival and *y*-axis is actual survival. The reference line is 45 degree and indicates perfect calibration.

To predict the OS and DFS of patients with CRC, two nomograms were established by multivariate Cox regression model according to all significantly independent factors for OS and DFS (Figure 2A, 2B). Nomograms can be interpreted by summing up the points assigned to each variable, which is indicated at the top of scale. The total points can be converted to predicted 5-year probability of death and recurrence or metastasis for a patient in the lowest scale [6, 17]. The Harrell’s c-indexes for OS and DFS prediction were 0.715 (95% CI: 0.683–0.748) and 0.704 (95% CI: 0.678–0.730), respectively. Calibration curves for two nomograms (Figure 2C, 2D) reveal no deviations from the reference line and no need of recalibration.

## DISCUSSION

Distant metastasis and local recurrence remain main concerns in patients with CRC [6, 18]. Factors known to be associated with decreased survival would provide the ability to pre-select those patients who would benefit most from more aggressive treatments or intensive follow-up [19]. Pathologic stage is valuable for predicting prognosis in patients with CRC; however, survival outcome is quite different even in patients at same stage. Although helical computed tomography and positron emission tomography may help to detect metastasis after radical surgery, for the potential radiation harm, they cannot be repeated performed, and the cost is also very high. Some oncogene or tumor suppressors were also proposed as valuable predictors, but most of these biomarkers were only validated in relative small number of patients, and some of them may difficult to measure due to sophisticated and expensive laboratory techniques were required.Identification of a simple and cost-effective indicator for predicting patient prognosis is an important goal.

Bile acid is the end product of cholesterol breakdown, which takes place in hepatocytes, and there are three formations in peripheral blood, TBIL, DBIL and IDBIL. In this study, we demonstrated that pretreatment serum DBIL was an independently prognostic factor for both OS and DFS in stage II and stage III CRC patients after surgery. High DBIL level correlated with lymph node metastases and lymphovascular invasion. To illustrate the improvement of the DBIL on the prediction of CRC survival, we developed two nomogram for OS and DFS included the traditional prognostic factors (T stage, N stage, lymphovascular invasion, perineural invasion, et al.) and DBIL, and found the nomogram could give a well prediction for survival. Another strength of our study is that we first treated TBIL, DBIL, IDBIL as a continuous variable, and validated DBIL as a potential predictor for both OS and DFS, then we used X-tile program, a robust graphical tool verified by Yale University [20], to verify the optimum cut-off values for DBIL. In fact, cut-off values of DBIL vary in different studies. For example, Gao et al. [8] used the thresholds 2.6 μmol/l as cutoff to classified the DBIL, while Li et al. [14] adopted 3.45 μmol/l as optimal cutoff in non-small-cell lung cancer, which value is quite close to our present study.It should be noted that in some studies used TBIL as main concern for survival analysis [15, 16, 21].TBIL consists of both DBIL and IDBIL, which may cause confuse.

As described in the background section, some epidemiological researches demonstrated that elevated serum bilirubin concentration was negatively correlated with the risk of CRC, whereas others found no significant association [13, 22]. However, different from their studies, our present study was designed to investigate the relationship between serum bilirubin and survival outcome in CRC. We found that increased serum DBIL was associated with a higher percentage of lymph node metastases and lymphovascular invasion, thus cause poor survival outcome, our conclusion also consisted with previous published articles [8]. Serum bilirubin levels also negatively correlated with response of metastatic CRC to irinotecan-based chemotherapy [23]. We hypothesis that serum bilirubin may play dual role in CRC tumorigenesis and progression.

Undeniably, there were some limitations in our study. First, we only included patients with stage II and III in the present study. Those metastatic patients and stage I CRC were not included. Thus the results cannot represent all patients with CRC. Second, only pretreatment DBIL was included in the study, and we cannot known the changes of DBIL in the course of therapy, and whether the changes may impact the survival outcome of CRC patients. Third, our study was complemented in two center cohorts included only Chinese patients. Although internal validation was performed to prevent over-interpretation of current data, it would be better is external validation can be carried out to verify whether our findings are universally applicable [24].

In conclusion, our retrospective study demonstrates that increasing pretreatment DBIL significantly correlated with poor outcomes in stage II and stage III CRC patients after surgical resection. This biomarker was directly derived from routine laboratory test of liver function, and can be easily applied in the clinical setting.

## MATERIALS AND METHODS

### Study population

The study included 986 patients with CRC who received surgical resection between January 2005 and December 2010. The inclusion criteria were: (1) age > 18 years old; (2) pathological diagnosis of CRC adenocarcinoma; (3) without distant metastasis (M1); (4) underwent surgical resection; (5) without preoperative anti-tumor therapy; (6) CRC was the only one primary or first of more than one primary; (7) laboratory tests were obtained before surgery with defined value; (8) information on OS and DFS available. The exclusion criteria were as follows: (1) patients diagnosed with hereditary non-polyposis CRC and familial adenomatous polyposis; (2) patients with hepatobiliary disease (including malignancy, gallstones, cirrhosis, hepatitis, or alcoholic liver disease, et al.) or hematological disease which may affect the bilirubin measurement. (3) patients that did not have complete clinical and pathological data; (4) patients with stage I CRC were also excluded from the study for the survival outcome were extremely good for them. 5-Fu or 5-FU derivate agents based adjuvant chemotherapy was recommended for stage II patients with high-risk characteristics and all stage III patients within 3 to 4 weeks after surgery. Ethical approval was obtained from the Ethical Committee and Institutional Review Board of The Fifth People’s Hospital of Shanghai, Fudan University.

The following parameters were included in the analysis: age, gender, tumor differentiation, tumor stage, node stage, the number of retrieval lymph nodes and positive lymph node count, lymphovascular invasion, perineural invasion, TBIL, DBIL, and IDBIL. All patients were restaged according to the 7th edition of the American Joint Committee on Cancer TNM classification. Patients with stages II of high risk and all stage III tumor received adjuvant chemotherapy with fluoropyrimidine-based regimens.

### Statistical analysis

Statistical evaluation was conducted with SPSS 22.0 (SPSS Inc., Chicago, IL, USA) and R 3.1.2 software (Institute for Statistics and Mathematics, Vienna, Austria). X-tile 3.6.1 [25] (Yale University, New Haven, CT, USA) was used to determine the optimal cut-off value for DBIL. The 5-year OS and DFS were estimated by the Kaplan-Meier method, and the difference of variables was compared using log-rank tests. Univariate analysis was used to examine the association between various prognostic predictors and OS, DFS. Significant prognostic predictors associated with OS and DFS were included to perform multivariate analyses by using the Cox proportional hazards model. *P* < 0.05 was considered statistically significant. All confidence intervals (CIs) were stated at the 95% confidence level.

Nomograms for possible prognostic factors associated with OS and DFS were established by R software, and the model performance for predicting outcome was evaluated by Harrell’s concordance index (c-index) [26, 27]. In addition to measuring discriminative capacity by c-index, each model was evaluated with calibration curve in which predicted outcomes versus observed outcomes are graphically depicted, which made it possible to conduct further comparison of accuracy in estimating prognosis.
